# VDR promotes pancreatic cancer progression in vivo by activating CCL20-mediated M2 polarization of tumor associated macrophage

**DOI:** 10.1186/s12964-024-01578-x

**Published:** 2024-04-10

**Authors:** Hengzhen Li, Yuli Ruan, Chao Liu, Xiaona Fan, Yuanfei Yao, Yisheng Dai, Yushuai Song, Dan Jiang, Ning Sun, Guangtao Jiao, Zhuo Chen, Shiheng Fan, Fanfei Meng, Huike Yang, Yanqiao Zhang, Zhiwei Li

**Affiliations:** 1 Department of Gastrointestinal Medical Oncology, Harbin Medical University Cancer Hospital, Harbin, China; 2 Heilongjiang Province Key Laboratory of Tumor Immunology, Harbin, China; 3 Shenzhen Engineering Center for Translational Medicine of Precision Cancer Immunodiagnosis and Therapy, Shenzhen, China; 4 Heilongjiang Province Key Laboratory of molecular Oncology, Harbin, China; 5 Department of Anatomy, Harbin Medical University, Harbin, China

**Keywords:** Pancreatic adenocarcinoma, Vitamin D receptor, CCL20, Macrophage

## Abstract

**Background:**

Activation of VDR pathway was a promising anti-tumor therapy strategy. However, numerous clinical studies have demonstrated the effect of activating VDR is limited, which indicates that VDR plays a complex role in vivos.

**Methods:**

We analyzed the TCGA database to examine the association between VDR expression and immune cell infiltration in pancreatic adenocarcinoma (PAAD). Western blot, ELISA, ChIP, and dual-luciferase reporter assays were performed to determine the mechanism of VDR regulating CCL20. Migration assay and immunofluorescence were used to investigate the role of CCL20 in M2 macrophage polarization and recruitment. We employed multiplexed immunohistochemical staining and mouse models to validate the correlation of VDR on macrophages infiltration in PAAD. Flow cytometry analysis of M2/M1 ratio in subcutaneous graft tumors.

**Results:**

VDR is extensively expressed in PAAD, and patients with elevated VDR levels exhibited a significantly reduced overall survival. VDR expression in PAAD tissues was associated with increased M2 macrophages infiltration. PAAD cells overexpressing VDR promote macrophages polarization towards M2 phenotype and recruitment in vitro and vivo. Mechanistically, VDR binds to the CCL20 promoter and up-regulates its transcription. The effects of polarization and recruitment on macrophages can be rescued by blocking CCL20. Finally, the relationship between VDR and M2 macrophages infiltration was evaluated using clinical cohort and subcutaneous graft tumors. A positive correlation was demonstrated between VDR/CCL20/CD163 in PAAD tissues and mouse models.

**Conclusion:**

High expression of VDR in PAAD promotes M2 macrophage polarization and recruitment through the secretion of CCL20, which activates tumor progression. This finding suggests that the combination of anti-macrophage therapy may improve the efficacy of VDR activation therapy in PAAD.

**Supplementary Information:**

The online version contains supplementary material available at 10.1186/s12964-024-01578-x.

## Introduction

Vitamin D receptor (VDR) is a member of the nuclear receptor family and regulates the expression of target genes by binding to a VDR element-containing promoter region [1]. VDR regulates cell proliferation, differentiation, apoptosis, autophagy, as well as the regulation of cell-microenvironment interactions such as epithelial-mesenchymal transition, angiogenesis, antioxidant function, inflammation, and immune system modulation [2–6]. Researchers widely believed that, activated VDR is a promising anti-tumor strategy with Vitamin D (VitD) serving as its activator [7]. Although intermittent VitD supplementation is currently favored in clinical studies, its efficacy in oncology remains controversial [8, 9]. When VitD supplementation was administered to pancreatic adenocarcinoma (PAAD) patients in clinical trials, it did not inhibit tumor size or improve patient prognosis [10, 11]. M. Waterhouse’s study even showed that the risk of PAAD increased with increasing dietary intake of VitD [12]. These findings indicate that the role of VitD/VDR in vivo is complex.

Mesenchymal tissue constitutes more than 80% of the tumor volume in PAAD. This primarily includes abundant extracellular stroma and immunosuppressive cells like tumor-associated macrophages (TAMs), myeloid-derived suppressor cells (MDSCs), cancer associated fibroblasts (CAFs), and regulatory T cells (Tregs) [13]. Previous study has reported an association between VDR and the regulation of immune cell function [14, 15]. Meanwhile, VDR expression in tumor cells and stroma was shown to be independent of patient prognosis in colorectal cancer (CRC). VitD-associated gene signature in CAFs correlated with longer overall survival (OS) and disease-free survival (DFS) in CRC [16]. However, the impact of high VDR expression in PAAD cells on the immune microenvironment remains unclear. Macrophages as the most abundant immune cells within the tumor [17], we hypothesized that PAAD cells overexpressing the VDR could promote more TAMs infiltration, thereby influencing the malignant evolution of PAAD.

Here, we analyzed the TCGA database and proved that the high expression of VDR in PAAD suggests poor prognosis. Increased expression of VDR in PAAD cells can promote macrophage M2 polarization and recruitment in vitro and vivo. Mechanistically, VDR up-regulates the expression of CCL20 to affect macrophages. The effects of polarization and recruitment on macrophages can be rescued by blocking CCL20. Concomitant administration of macrophage-scavenging agents for patients with VDR-overexpressing PAAD or supplemental VitD therapy may provide new ideas for the treatment of PAAD patients.

## Materials and methods

### Patients and samples

In this study, we collected tissues from 87 patients with PAAD who attended the Harbin Medical University Cancer Hospital from 2011 to 2018 for immunohistochemical analysis. All patients have not recepted radiotherapy and neoadjuvant chemotherapy, and all patients received follow-up until November 2019. The patients provided signed informed consent forms and were approved by the Ethics Committee of Harbin Medical University Cancer Hospital (KY2022-38).

### Cell culture and viral infection

Human pancreatic ductal epithelial HPDE6-C7 and human PAAD cell lines (PL-45, Capan1, BxPC3, PSN1, Miapaca2, Panc1, SW1990) were purchased from American Type Culture Collection. THP-1 was purchased from Pricella company in Wuhan. Cells were cultured in RPMI 1640 (HPDE6-C7, PSN1, BxPc3, THP-1), DMEM (SW1990, Panc1, PL45, MiaPaCa2, Pan02) and IMDM (Capan1) medium. All cell lines were supplemented with 10% fetal calf serum, streptomycin (100U/mL), and penicillin (100U/mL), and were maintained in a humidified atmosphere of 5% CO_2_ at 37 °C. All cell lines underwent validation using STR DNA fingerprinting. Experiments were conducted within six months of obtaining the cell lines. Additionally, mycoplasma contamination was ruled out using a PCR-based method.

Panc1 cells were used for VDR overexpression, while Capan1 cells were used for knockdown. The VDR (Homo sapiens) ORF sequences were cloned into pCDH-EF1-MCS-CMV-copGFP-T2A-Puro lentiviral vectors (Symbio Technologies, China), and VDR shRNA was constructed into pLent-U6-shRNA-cmv-copGFP-P2A-Puro (Vigene Biosciences, China). The VDR (Mus) ORF and CCL20(Mus) ORF sequences were cloned into pCDH-CMV-MCS-EF1-Puro lentiviral vectors, and VDR (Mus) shRNA and CCL20 (Mus) shRNA was constructed into pLKO.1-puro (Symbio Technologies, China). Subsequently, the lentiviral vectors were used to package the viral particles. Following that, the Panc1 and Capan1 cell lines were infected with the virus for 24 h and selected with puromycin (1 µg/mL, Sigma-Aldrich, USA) for 1 week.

### Macrophage differentiation and cell co-culture

THP-1 cells were used to construct a model of macrophage polarization. THP-1 cells were differentiated into macrophages (M0 macrophage) using 100 ng/mL of phorbol 12-myristate 13-acetate (PMA, Abcam, USA) for 24 h. Macrophages were co-cultured with PAAD cell lines condition medium with IgG antibody (15 ng/ml; Abcam, USA) or anti-CCL20 antibody (15 ng/ml; Abcam, USA). After 48 h of co-culture, the differentiated macrophages were harvested for further experiments. The M0 macrophages were polarized into M2 macrophages via 20 ng/ml IL-4 (Peprotech, USA) and 20 ng/ml IL-13 (Peprotech, USA) stimulation for 48 h.

### **Chromatin immunoprecipitation** (**ChIP)**

Panc1 cells (3 × 10^6^) were seeded in 100 mm dishes, and after treatment with 1% formaldehyde, the cells were lysed using 600 µl of radioimmunoprecipitation assay (RIPA) lysis buffer. Genomic DNA was isolated and fragmented into 200–600 bp fragments using a sonicator. Following centrifugation, the supernatants were collected and chromatin was incubated and precipitated with antibodies recognizing VDR (1:50, abcam, USA) or IgG (Beyotime Biotechnology, China) at 4 °C overnight. The immune complexes were then precipitated using protein A/G-PLUS-Agarose beads (SANTA CRUZ, USA) for 4 h. Subsequently, the immune complexes were washed with different washing buffers, including a low salt washing buffer, high salt washing buffer, LiCl washing buffer, and TE buffer. The immune precipitates were eluted using 500 µl of elution buffer and cross-linking was reversed by incubating at 65 °C overnight. The binding sites for VDR were amplified using CCL20 promoter primers.

### Flow cytometry assay for immune cell infiltration

The subcutaneous graft tumors were minced with clippers, and collagenase was added before being placed on a shaker at 37˚C for digestion. Filtration was performed using filters, and the resulting filtrate was then centrifuged at 2000 rpm for 5 min. The supernatant was discarded, and the remaining cells were treated with erythrocyte lysate for 5 min. After that, the cells were washed with PBS. Flow antibodies (Biolegend, USA) were added and the mixture was incubated for 30 min at 4˚C, protected from light. Following the incubation, the cells were washed with PBS and then resuspended for cell ratio analysis using FlowJo software.

### Xenograft assays

All animal experiments were approved by the Committee of Experimental Animals of Harbin Medical University and complied with the Regulations for the Administration of Affairs Concerning Experimental Animals. Six-week-old female mice (C57-BL6J) (Vital River Laboratories, China) were housed under controlled light conditions. A total of 2 × 10^6^ Pan02 cells in 0.1 ml PBS were injected into the right flank of the mice. In the CCL20 blockade assay, seven days after cell implantation, anti-CCL20 antibody (1 mg/kg) or IgG antibody (1 mg/kg) as a control was administered locally to the mice every 2 days for 4 weeks. Tumor volume (V) was calculated using the following formula: (width) ^2^ × height/2. The mice were euthanized 4 weeks after tumor implantation.

### Multiplexed immunohistochemical (mIHC)

Tissue microarrays (Shanghai Outdo Biotech CO.LTD) were deparaffinized using xylene and a gradient ethanol solution. Antigen repair was performed by heating with antigen repair solution. Endogenous peroxidase activity was neutralized with an endogenous peroxidase blocking solution, and the binding of irrelevant antibodies was blocked with BSA. Primary antibodies used in this study included VDR, CD163, CD86, CD68, CCL20, and PanCK. Following primary antibody staining, tissues were stained with Opal polymerized HRP anti-mouse/rabbit secondary detection antibodies. Tissues were then incubated with one of the following fluorophores, Opal Polaris 520, Opal Polaris 570, Opal Polaris 620, Opal Polaris 690, or Opal Polaris 780, according to the manufacturer’s instructions (dilution 1:100). Tissue microarrays were mounted in ProLong Gold antifade reagent with DAPI. Whole tissue sections were scanned at 20x magnification using a Vectra Polaris system (Akoya Biosciences, USA) to capture the stained images. To analyze the spatial distribution of CD68+, CD163+, and CD86 + cells in the area surrounding the tumor, an algorithm was designed to create a 200 μm-thick band outside the tumor margin. The peritumoral compartment was defined as the region outside the tumor margin within 200 μm.

### Analysis based on TCGA databases

We utilized R software to analyze the expression levels of VDR in PAAD tissues and normal tissues from the TCGA and GTEx databases. The optimum cut-off point for determining the patients classification of survival subgroups was calculated using the “surv cutpoint” algorithm of the “survival” R package. Univariate and multivariable analyses were conducted using Cox proportional hazards regression models. Multivariable analyses were used stepwise regression analysis. The ssGSEA algorithm was used to evaluate the infiltration abundance of 28 kinds of immune cells in TCGA PAAD data set. In addition, we further validated the correlation between analyzing VDR and immune cell infiltration by TIMER2.0 website. Enrichment of immune-related pathways by KEGG for differential genes between high and low VDR expression groups.

### Statistical analysis

Statistical analysis was performed using GraphPad Prism version 9.0 (GraphPad software, USA). The measurement data are presented as mean ± standard deviation (SD). An independent samples t-test was used to analyze the differential expression levels of VDR mRNA between the PAAD tissues and the adjacent normal tissues from TCGA databases. Differential analysis was conducted using paired t-tests after quantifying immunofluorescence in 19 pancreatic carcinomas and their paired paracancerous tissues. Correlations between VDR expression and clinicopathological characteristics were analyzed using Pearson’s Chi-squared test. Overall survival (OS), progression free interval (PFI), disease free interval (DFI) and disease-specific survival (DSS) analysis were performed using Kaplan-Meier plots, and the differences were compared using the log-rank test. A two-tailed *P*-value of 0.05 was considered statistically significant. * means *P* < 0.05; ** means *P* < 0.01, *** means *P* < 0.001.

## Result

### VDR is over-expression in PAAD and is associated with poorer prognosis

**Fig. 1:**
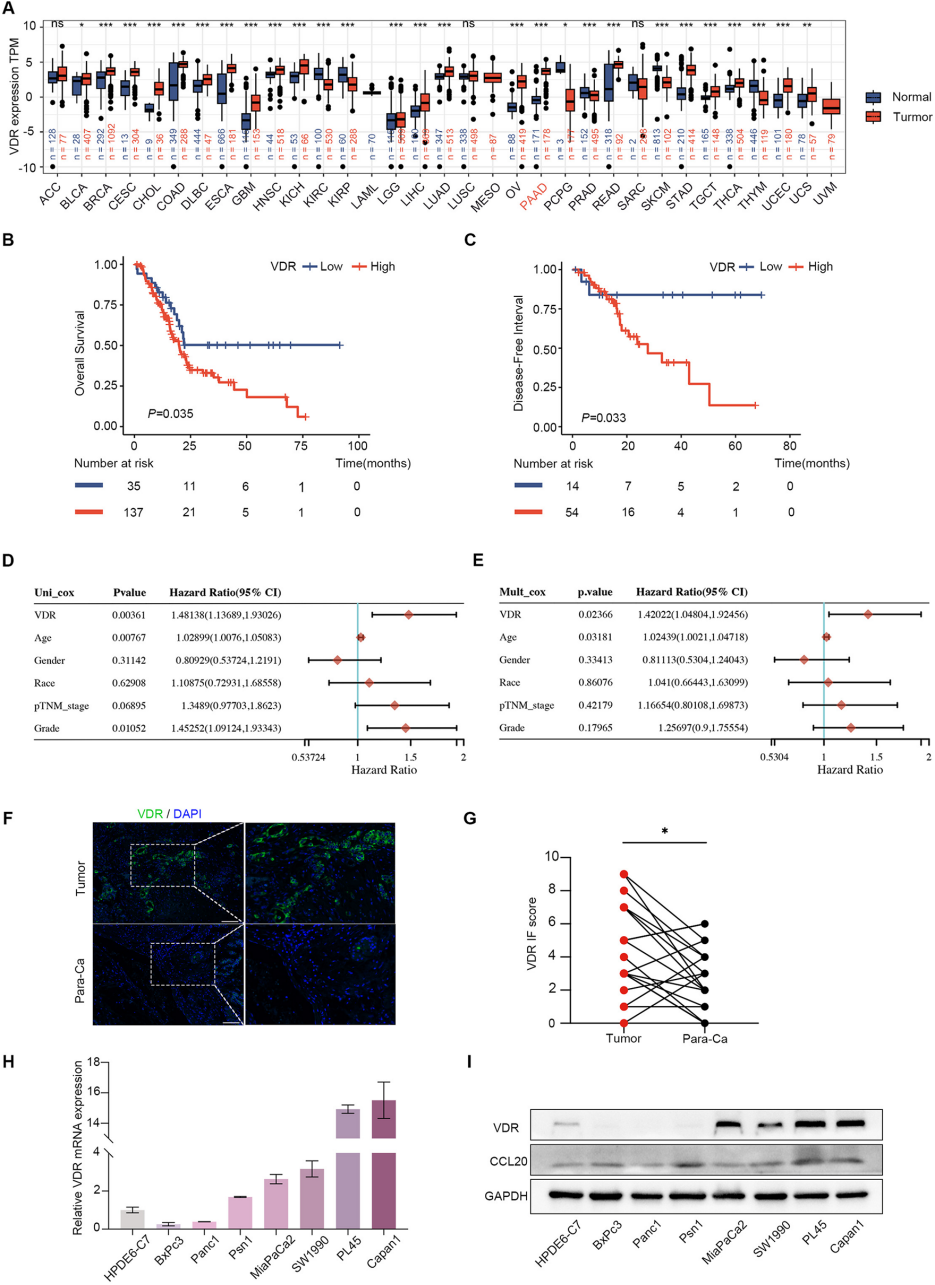
Elevated expression of VDR in PAAD. **(A)** The level of VDR expression in different tumor types from TCGA and GTEx databases. **(B-C)** OS and DFI of VDR high and low PAAD patients analyzed by TCGA data. **(D-E)** Univariate and Multivariate Cox analysis showing the hazard ratios (HRs) of different factors. **(F-G)** The relative expression of VDR in 19 cases of PAAD and paired paracancerous tissues was detected using immunofluorescence. Scale bars = 50 μm. (*P* < 0.05). **(H-I)** The expression of VDR and CCL20 was compared among different PAAD cell lines. The values in the graphs represent the mean ± SD. * means *P* < 0.05; ** means *P* < 0.01, *** means *P* < 0.001

We analyzed the mRNA expression of VDR in various human tumors using the TCGA database and discovered that VDR was highly expressed in PAAD and multiple tumors (Fig. 1A). Although, there were no significant differences in VDR expression among different grade, stage, and TNM staging subgroups in PAAD (Supplementary Fig. 1A-E). The KM survival curve indicated that high VDR expression was associated with poorer OS (Fig. 1B) and DFI (Fig. 1C). Univariate and multivariate analyses demonstrated that VDR expression and age are independent adverse prognostic factors (Fig. 1D and E). Moreover, high expression of VDR was associated with a poorer prognosis in various age, grade, and stage subgroups (Supplementary Fig. 1F-Q). However, our in vitro experiments demonstrated that VDR inhibited the proliferation and migration of PAAD cells (Supplementary Fig. 2A-H). We performed immunofluorescence staining of 19 cases of PAAD and paired paracancerous tissues and found that VDR expression was higher in tumor tissues (Fig. 1F-G). We validated VDR expression on cell lines and demonstrated low expression in the normal pancreatic cell line HPDE6-C7 (H6C7) and high expression in MiaPaCa2, SW1990, PL45 and Capan1 (Fig. 1H-I).

### VDR is associated with M2-macrophages infiltration in PAAD

**Fig. 2:**
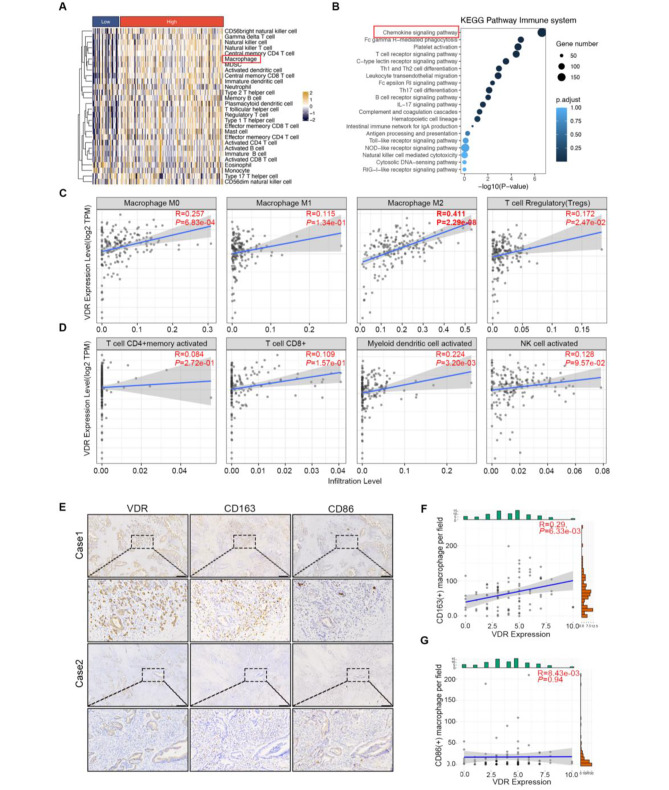
VDR is associated with M2 macrophages infiltration in PAAD. **(A)** Heatmap of VDR expression in PAAD correlating with infiltration of 28 immune cells. **(B)** KEGG analysis revealed the top 20 immune system pathways that are significantly different between the VDR high and low groups. **(C-D)** The TIMER2.0 database system was used to analyze the correlation between VDR expression and immune cells infiltration in PAAD. **(E)** Immunohistochemistry was performed to detect the expressions of VDR and macrophages in PAAD. Scale bars = 300 μm. **(F-G)** Pearson correlation analysis was used to assess the correlation between VDR expression and macrophages infiltration

We analyzed the infiltration of 28 immune cells in PAAD patients of TCGA database using the ssGSEA algorithm and generated heat maps. Significant correlations were found with several immune cells, including macrophages, dendriticcells (DCs), and MDSCs (Fig. 2A). KEGG pathway analysis of the immune system revealed the highest enrichment in the chemokine signaling pathway (Fig. 2B). Additionally, we utilized the TIMER2.0 website to assess the relationship between VDR expression and the predominant immune cells in tumors. Our correlation analysis indicated that VDR was positively correlated with macrophages infiltration, particularly M2 macrophages infiltration, while no correlation was observed with infiltration of M1 macrophages (Fig. 2C). Moreover, VDR expression in PAAD was associated with Treg and myeloid DCs infiltration, independent of CD4 + T cells, CD8 + T cells, and NKs. We collected 87 cases of PAAD pathological tissues from Harbin Medical University Cancer Hospital (Table 1) and conducted immunohistochemical staining for VDR, the M2 marker CD163, and the M1 marker CD86 (Fig. 2E). Correlation analysis revealed an association between VDR and M2 macrophages infiltration (Fig. 2F), while no significant correlation was found with M1 macrophages infiltration (Fig. 2G).

**Table 1:** Correlation between VDR expression and clinicopathological characteristics in PAAD patients

Characteristics	VDR		P.overall
	High (*n* = 66)	Low (*n* = 21)	
Gender			0.129
Female	23 (34.8%)	3 (14.3%)	
Male	43 (65.2%)	18 (85.7%)	0.650
Age(years)	56.3	55.1	
Tumor location			0.880
Head	41 (62.1%)	12 (57.1%)	
Neck/Body/Tail	25 (37.9%)	9 (42.9%)	
Differeation			0.420
High	20 (30.3%)	4 (19.0%)	
Low	9 (13.6%)	5 (23.8%)	
Moderate	37 (56.1%)	12 (57.1%)	
T stage			0.024*
1–2	9 (13.6%)	8 (38.1%)	
3–4	57 (86.4%)	13 (61.9%)	
N stage			0.336
0	50 (75.8%)	13 (61.9%)	
1	13 (19.7%)	6 (28.6%)	
M stage			0.352
0	62 (93.9%)	18 (85.7%)	
1	4 (6.06%)	3 (14.3%)	

### VDR overexpression tumor cells promote the macrophages polarization and recruitment

**Fig. 3:**
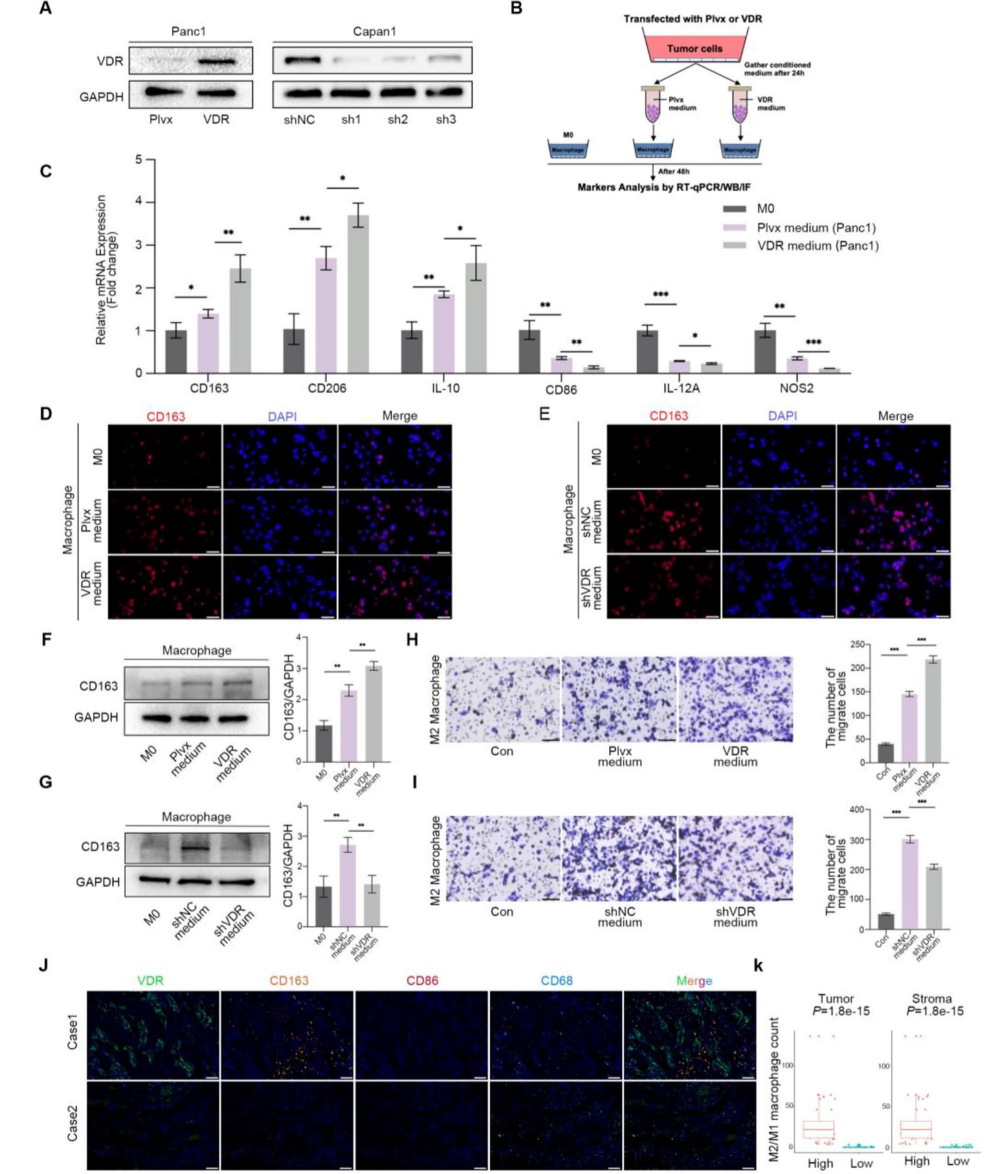
VDR overexpression tumor cells promote the macrophages polarization and recruitment. **(A)** Construction of recombinant cell lines overexpressing and knocking down VDR was achieved using lentivirus in Panc1 and Capan1, respectively. **(B-G)** qPCR, immunofluorescence, and western blot were performed to measure the expression of M1 and M2 markers after co-culturing with transfected PAAD cells. Scale bars = 50 μm. **(H-I)** Representative images of macrophage migration assays and quantitative chart. Scale bars = 200 μm. **(J)** mIHC was performed on PAAD tumors from TMA. Scale bars = 100 μm. The values on the graphs represent are mean ± SD. Statistical significance was determined as *P < 0.05, **P < 0.01, ***P < 0.001

As shown in Fig. 1I, Capan1 highly expressed VDR, while Panc1 expressed VDR at a low level. Therefore, we selected Panc1 to overexpress VDR and Capan1 to knock down VDR (Fig. 3A). The conditioned medium was collected and co-cultured with M0 macrophages (Fig. 3B). After 48 h, the expression of M2 markers CD163, CD206 and IL-10 significantly increased in the conditioned medium with VDR overexpression, while M1 markers CD86, IL-12 A and NOS2 were significantly inhibited (Fig. 3C). Immunofluorescence analyses showed a significant increase of CD163 positive macrophages (Fig. 3D), which was reduced by treatment with conditioned medium from Capan1 with VDR knockdown (Fig. 3E). The alterations in CD163 expression were also confirmed through western blot (Fig. 3F and G). To investigate the relationship between intra-tumor VDR and M2 macrophages recruitment, we performed macrophages migration assay. The results confirmed that conditioned medium from Panc1 cells, which are positive for VDR, can promote M2 macrophages recruitment (Fig. 3H). This effect can also be eliminated by knockdown VDR in Capan1 cells (Fig. 3I). The findings suggest that VDR overexpression in conditioned medium stimulates M2 polarization and recruitment. Additionally, utilizing multiplexed immunohistochemistry (Fig. 3J), we observed increased infiltration of both M2 and M1 macrophages in tumor tissue and stroma (Supplementary Fig. 3A-D). We confirmed that tumor tissues overexpressing VDR contained a higher ratio of M2/M1 macrophages in both the tumor and stroma (Fig. 3K).

### VDR overexpression in tumor cells promotes CCL20 transcription and release

**Fig. 4:**
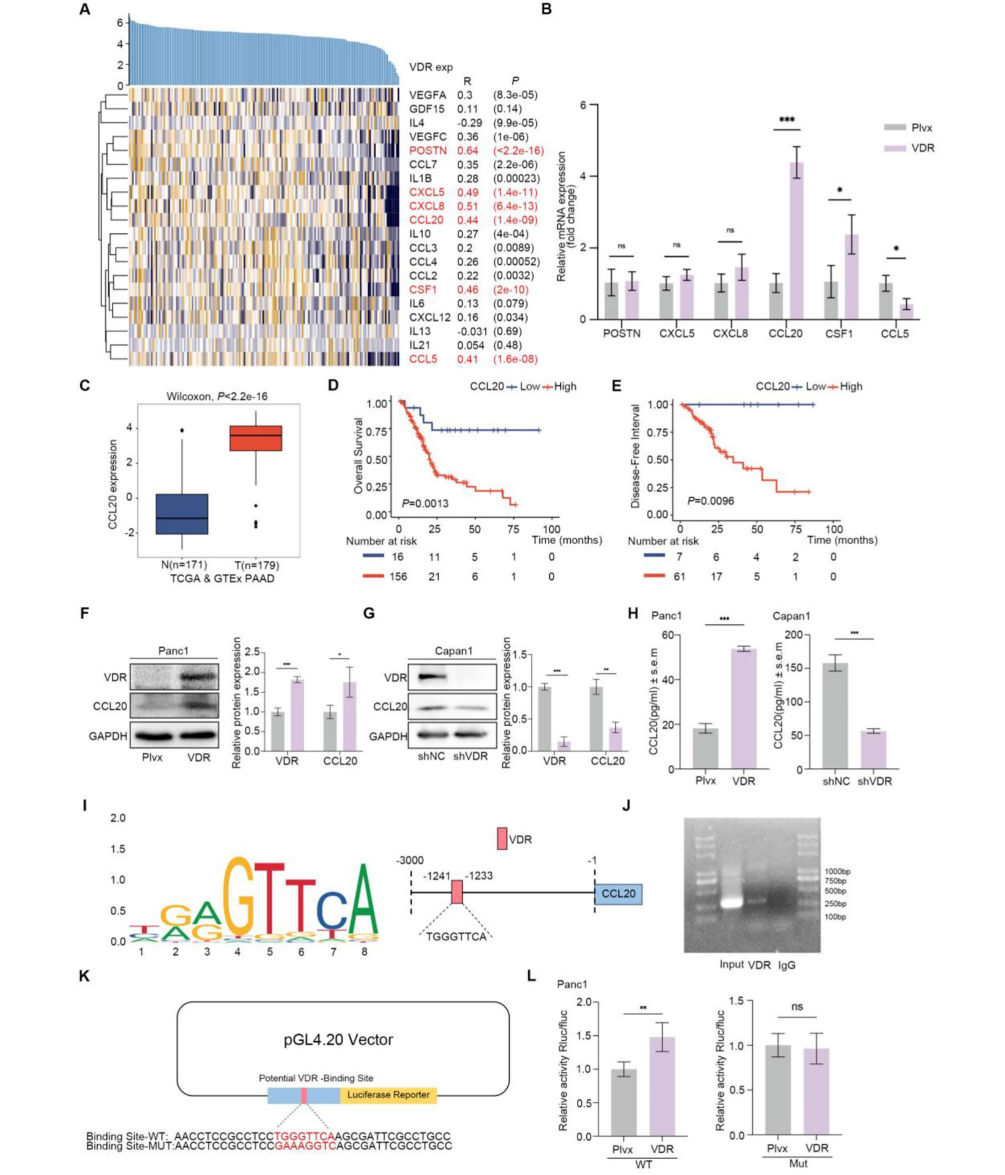
VDR promotes the transcription and release of CCL20. **(A)** Heat map displayed the correlation between VDR and macrophage M2 polarization and recruitment gene set in TCGA PAAD data set. **(B)** Gene expression validation by qPCR. **(C)** The expression of CCL20 between normal and PAAD tissues. **(D-E)** Kaplan-Meier curves for CCL20 high and low groups. **(F-G)** The expressions of VDR and CCL20 detected by western blot. **(H)** Measurement of CCL20 concentration in the supernatants of VDR overexpression and VDR knockdown tumor cells by ELISA. **(I)** Prediction of VDR and CCL20 binding sites. **(J)** ChIP assay confirmed that VDR could bind to the CCL20 promoter in Panc1. **(K)** Schematic representation of the CCL20 promoter and its mutant site luciferin-reporter gene. **(L)** Luciferase assay demonstrated that VDR promoted CCL20 transcription. Values in the graphs are presented as mean ± SD. *P < 0.05, **P < 0.01, ***P < 0.001

We analyzed the correlation between VDR and chemokines involved in macrophage polarization and recruitment using data from TCGA PAAD date set (Supplementary Fig. 4A-T). Our analysis revealed a strong correlation between VDR expression with the POSTN, CXCL5, CXCL8, CCL20, CSF1 and CCL5 (Fig. 4A). To validate this correlation, we performed qPCR and observed a significant increase in CCL20 expression in Panc1 cells overexpressing VDR (Fig. 4B). Furthermore, through TCGA data, we confirmed that CCL20 is highly expressed in PAAD (Fig. 4C) and is associated with poorer OS and DFI (Fig. 4D and E). We also validated the correlation between VDR and CCL20 at the protein level (Fig. 4F-G). Subsequently, we conducted an ELISA analysis and confirmed that CCL20 levels were elevated in the supernatant after VDR overexpression (Fig. 4H). We also observed a correlation between the concentration of CCL20 and the level of VDR protein in PAAD cell lines (Fig. 1I, Supplementary Fig. 5A-B). We stimulated Panc1 cells with the VDR agonist calcitriol (Cal), which resulted in the activation of the VDR. Subsequently, the mRNA and protein levels, as well as the secretion of CCL20, were significantly increased (Supplementary Fig. 5C-E). As mentioned earlier, VDR acts as a transcription factor to regulate transcription of downstream gene promoters. Therefore, we utilized the JASPAR website to predict the possible binding sites of VDR on the CCL20 promoter (Fig. 4I). Subsequently, we performed a chromatin ChIP assay to demonstrate VDR binding to the CCL20 promoter region (Fig. 4J). To investigate the binding of the VDR to the CCL20 promoter site and its effect on transcriptional activation, we assessed the influence of a luciferase reporter containing either the wild-type (Site-WT) or mutant (Site-Mut) VDR-binding sites within the CCL20 promoter (Fig. 4K). Transcriptional activation by the VDR at the promoter sites was abolished following mutation of the CCL20 promoter (Fig. 4L).

### Blocking CCL20 rescues VDR-mediated macrophage polarization and infiltration

**Fig. 5:**
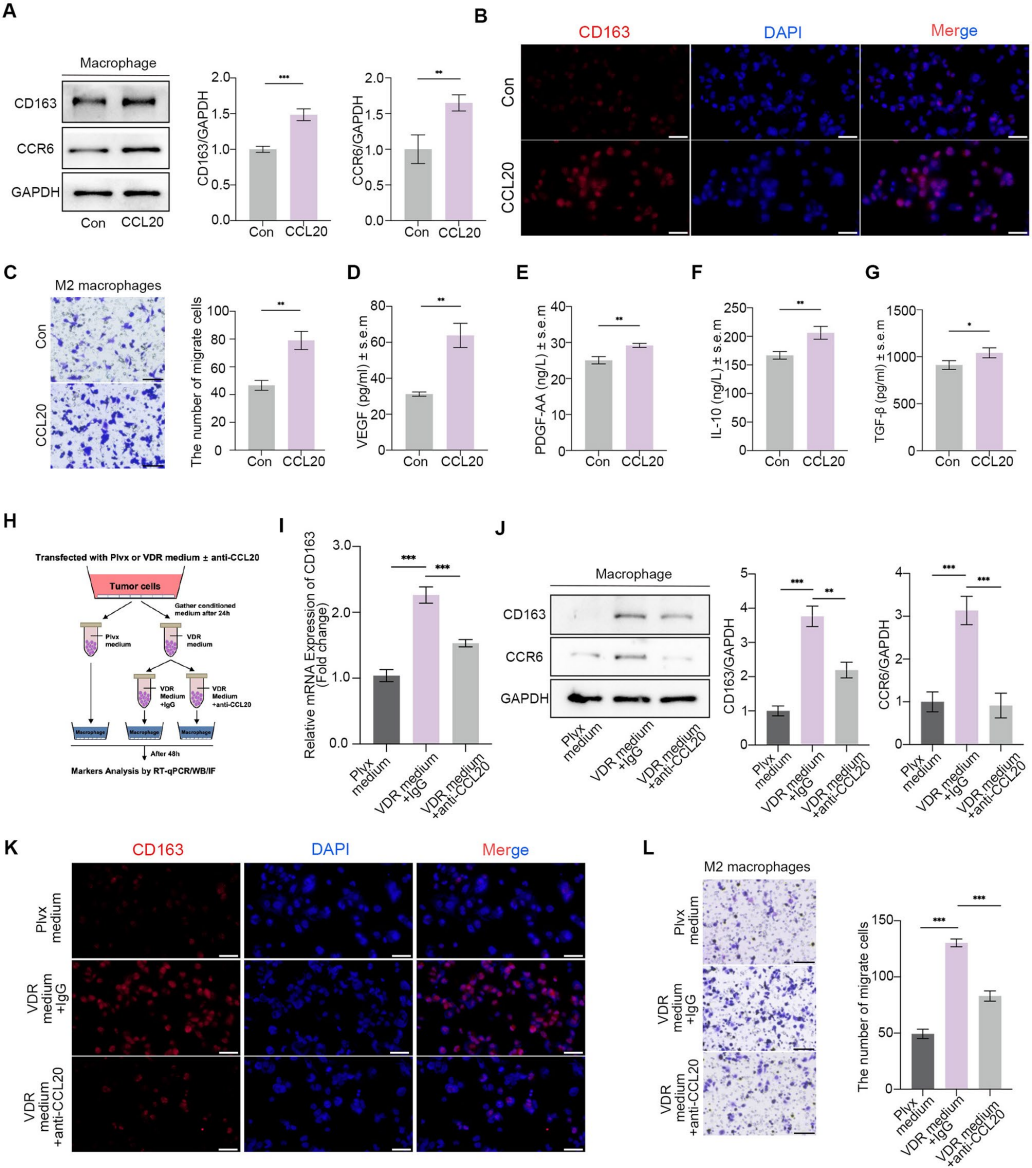
Blocking CCL20 rescues VDR-mediated macrophages polarization and infiltration. **(A-B)** Western blot and immunofluorescence results of CD163 following stimulation of macrophages with CCL20. **(C)** Representative images showing the influence of CCL20 on M2 macrophages migration assays. **(D-G)** ELISA assays measuring the secretion of VEGF, PDGF-AA, IL-10, and TGF-β by M2 macrophages. **(H-K)** CD163 was tested using qPCR, immunofluorescence, and western blot after co-culturing with condiction medium pretreated with IgG or anti-CCL20 antibody. Scale bars = 200 μm. **(L)** Representative images of M2 macrophages migration assays induced by condition medium. Scale bars = 200 μm. Values presented in the graphs represent mean ± SD. *P < 0.05, **P < 0.01, ***P < 0.001

We initially validated the impact of CCL20 on macrophage polarization and recruitment. CCL20 notably induced the expression of the M2 macrophage marker CD163 (Fig. 5A and B). Additionally, CCL20 is the only known high-affinity ligand that binds to CCR6 and promotes macrophage polarization and migration. In our study, we found that CCL20 activated CCR6 on macrophages (Fig. 5A). Furthermore, CCL20 facilitated the recruitment of M2 macrophages (Fig. 5C). Macrophages significantly increased secretion of VEGF, PDGF-AA, IL-10, and TGF-β after stimulation with CCL20 (Fig. 5D-G). M2 macrophages play a role in promoting tumor angiogenesis and the immunosuppressive microenvironment through the secretion of these cytokines, further validating that macrophages undergo M2 polarization. We conducted revertant experiments by blocking CCL20 with a neutralizing antibody before stimulating M0 with conditioned medium (Fig. 5H). Initially, CCR6 expression on macrophages was attenuated, and CD163 expression was reduced after blocking CCL20 in the conditioned medium, suggesting that blocking CCL20 in VDR overexpression conditioned medium reduced M2 polarization in macrophages (Fig. 5J and K). Additionally, the recruitment of M2 macrophages by the conditioned medium was also significantly reduced after blocking CCL20 in the conditioned medium (Fig. 5L). In summary, CCL20 is a key cytokine in VDR influencing macrophages function.

### VDR correlates with CCL20 expression and macrophages infiltration in PAAD

**Fig. 6:**
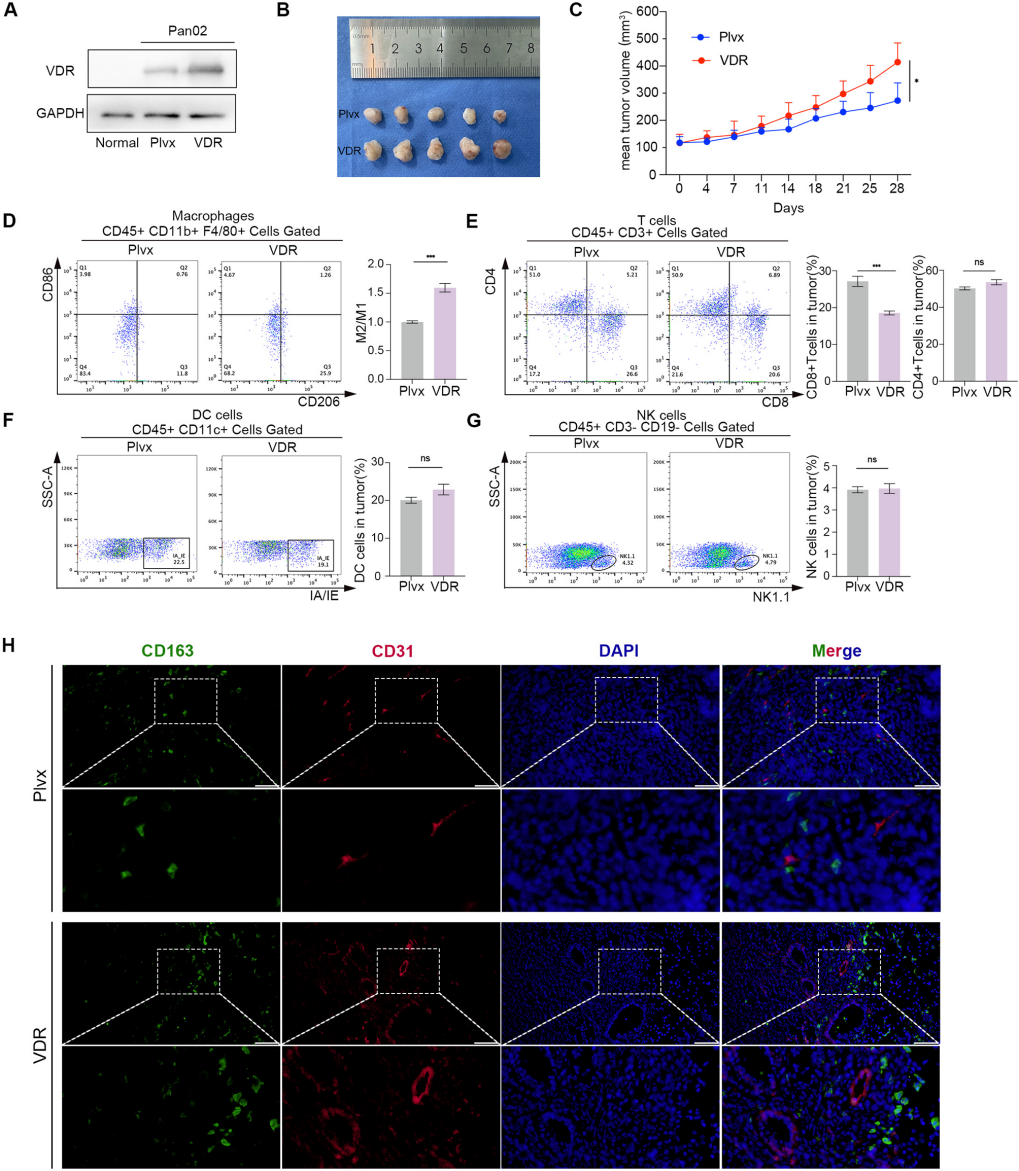
High VDR expression correlates with increased M2 macrophages infiltration in vivo. **(A)** Protein expression levels of VDR in normal pancreatic tissues, Pan02 cell line, and the overexpressed VDR Pan02 cell line. **(B)** Subcutaneous graft tumors derived from the Plvx group or the VDR group. **(C)** The volumes of subcutaneous graft tumors were assessed twice a week. **(D-G)** Staining and flow cytometric analysis of macrophages, T cells, DCs and NKs infiltration across different VDR expression groups. **(H)** Immunofluorescence co-staining was performed to detect the M2 macrophages and vascular endothelial. Values presented in the graphs represent mean ± SD. *P < 0.05, **P < 0.01, ***P < 0.001

In order to characterize resident and infiltrating macrophages in PAAD, we discovered that the expression of VDR in Pan02 cells was higher than in normal pancreatic tissues of C57BL6J mice (Fig. 6A). Additionally, we successfully established a cell line in which Pan02 cells stably overexpressed VDR. The tumor volume of overexpressing VDR was significantly larger than the control group (Fig. 6B and C), while flow analysis showed a higher number of M2/M1 macrophages infiltration than the control group (Fig. 6D). Subsequently, we examined the infiltration of CD4 + T cells, CD8 + T cells, DCs, and NKs within the tumor tissues using flow cytometry. The infiltration of CD8 + T cells was reduced in the group with VDR overexpression (Fig. 6E), while there was no significant change in the proportion of CD4 + T cells, DCs, and NKs infiltration (Fig. 6E-G). Additionally, through co-staining CD163 and the vascular endothelial cell marker CD31 with immunofluorescence (Fig. 6H), we found a significant increase in angiogenesis following VDR overexpression, as well as an increase in M2 macrophages infiltration.

### CCL20 is a crucial cytokine that enables VDR to promote tumor proliferation in vivo

**Fig. 7:**
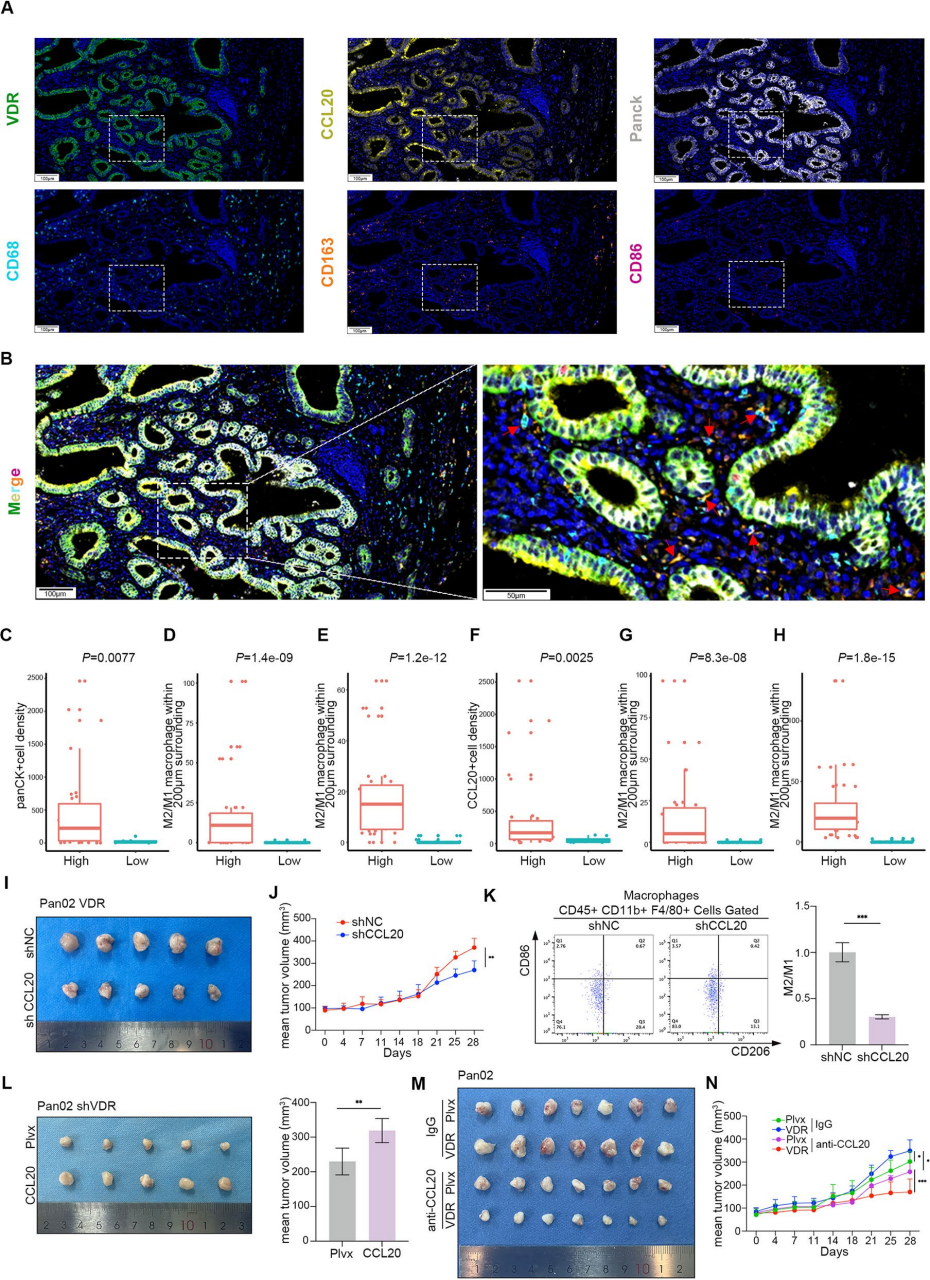
High VDR expression correlates with increased M2 macrophages infiltration in tumor tissues. **(A-B)** Representative composite image of a TMA core with Opal six-color multiplex staining. Pseudocolor illustrating CD68 (cyan), VDR (green), Panck (white), CCL20 (yellow), CD86 (red), CD163 (orange), and DAPI (blue) staining. Scale bars = 100 μm. **(C-F)** Comparison between different VDR expression groups of panCK + cell density, CCL20 + cell density, M2/M1 macrophage within 200 μm of the VDR positive cells in tumor and stroma. **(G-H)** Comparison between different CCL20 expression groups of M2/M1 macrophage within 200 μm of CCL20 positive cells in tumor and stroma. **(I)** Subcutaneous graft tumors interfering with CCL20 expression in the Pan02 cell line overexpressing VDR. **(J)** Proliferation curve of tumor volume. **(K)** Staining and flow cytometric analysis of M2/M1 infiltration across different CCL20 expression groups. **(L)** Subcutaneous tumors after intervention of CCL20 expression on Pan02 with knockdown of VDR expression. **(M)** Subcutaneous graft tumors after treatment with local injections of IgG and CCL20 antibodies administered in the Plvx control and VDR overexpression groups, respectively. **(N)** Proliferation curve of tumor volume. Values presented in the graphs represent mean ± SD. *P < 0.05, **P < 0.01, ***P < 0.001

We established and optimized a workflow for mIHC to assess the expression of VDR, CCL20, PanCK, CD68, CD163, and CD86. Following spectral separation using inForm software, the images were divided into their respective fluorophores, and the corresponding images were visualized (Fig. 7A-B). We performed a 6-color mIHC staining on tissue microarray (TMA) samples to evaluate the tumor area and the densities of VDR overexpression cells, CCL20 overexpression cells, M2 macrophages and M1 macrophages. Initially, we observed that the VDR high-density group exhibited a higher density of panCK + cells (Fig. 7C). There were higher M2/M1 ratios in the 200 μm range of VDR-positive cells both in the tumor and stroma (Fig. 7D and E). Additionally, upon more extensive analysis, a significant correlation was identified between VDR expression and CCL20 expression (Fig. 7F), with CCL20-positive cells also being surrounded by higher M2/M1 ratios in the 200 μm of tumor and stroma (Fig. 7G-H). In the Pan02 cell line overexpressing VDR, knockdown of CCL20 resulted in a deceleration of tumor cell proliferation in vivo and a reduction in the M2/M1 ratio (Fig. 7I-K). Conversely, in the Pan02 cell line with knockdown of VDR, the rate of tumor cell proliferation in vivo was partially restored following overexpression of CCL20 (Fig. 7L). Additionally, when CCL20 was administered in vivo, it exhibited a tumor inhibitory effect compared to the IgG group. Specifically, in the VDR overexpression group, the most substantial inhibitory effect on tumors was observed after CCL20 was blocked (Fig. 7M-N).

**Fig. 8:**
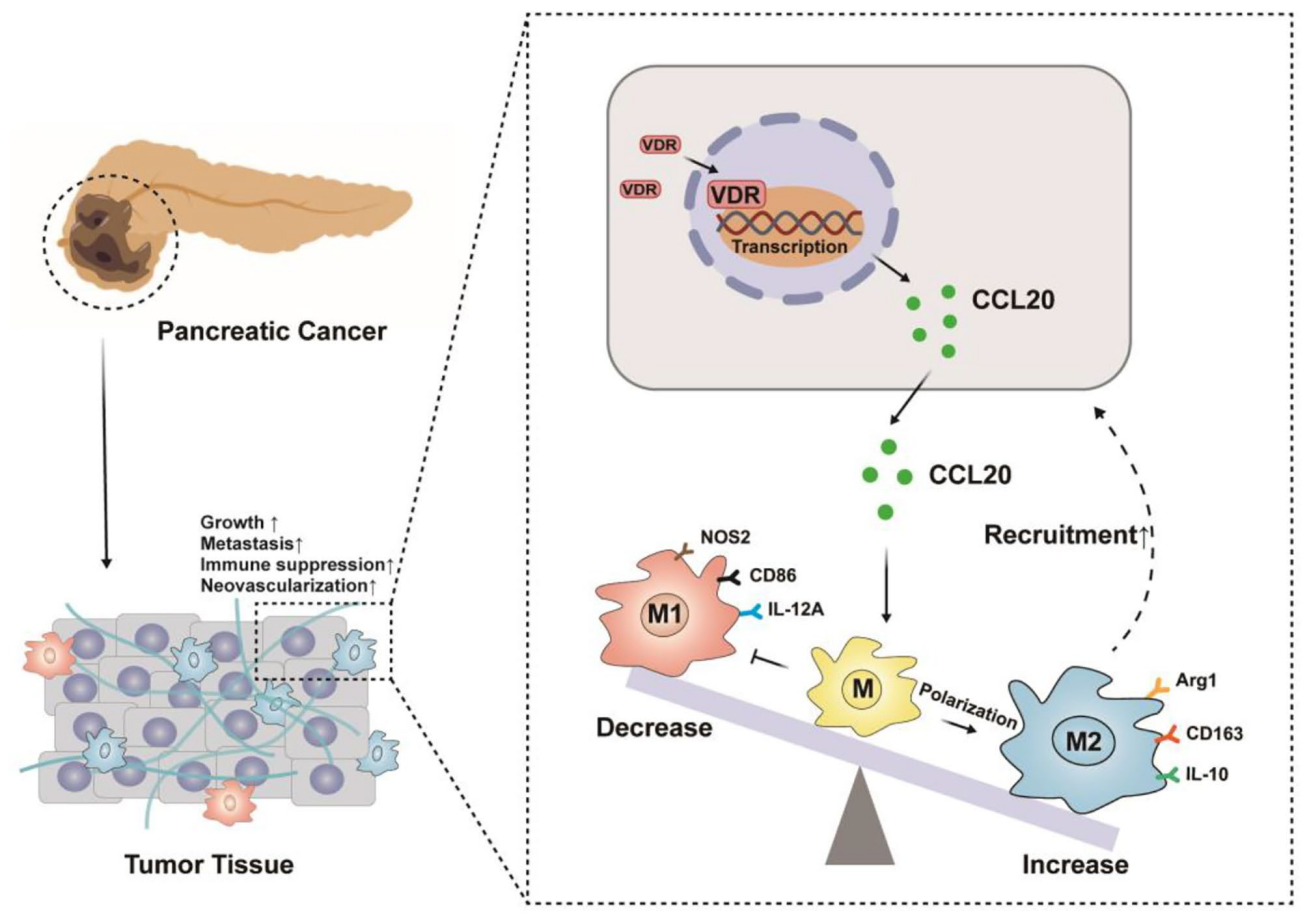
Interaction between tumor and macrophages in TME.

## Discussion

In recent years, there have been clinical studies examining the use of VitD to activate VDR in various tumors, such as colorectal cancer, ovarian cancer, breast cancer, and prostate cancer [18–21]. While VitD has shown promising anti-tumor effects in basic research [4, 22], clinical studies have not yet yielded the expected results [23]. On the one hand, this could be attributed to the specific acidic environment within the tumor, as evidenced in a recent study on CRC. The study revealed a new mechanism by which acidosis can impact the stemness of CRC cells through the regulation of SOX2 expression. Additionally, the study showed that aberrant VDR expression hinders the effective activation of VitD signaling, leading to a lack of efficacy in the antineoplastic process of VitD [18]. On the other hand, the prognostic significance of VDR expression in tumor cells and stroma variest. VDR expression in CAF of intestinal cancer tissue stroma is more associated with prognosis. Our previous published studies have shown that VDR overexpression in PAAD cells inhibits stemness and sensitizes cancer cells to gemcitabine [24, 25]. Subsequent clinical trials on VDR have revealed that VitD supplementation does not reduce mortality related to tumors or cardiovascular diseases. This implies that utilizing this treatment approach may not be effective in hindering tumor progression. Our study found that VDR expression was linked to a poor prognosis in patients with PAAD. This further emphasizes the need for caution when considering the use of VitD activated VDR anti-tumor supplementation in clinical settings.

It has been reported that VDR is involved in immunomodulatory processes. In terms of innate immunity, VitD promotes the production of cathelicidin and β-defensin 2, enhances autophagy through Toll-like receptor activation, and impacts complement concentration [26]. In adaptive immunity, VitD inhibits the maturation of dendritic cells and hinders antigen presentation. Additionally, VitD increases the production of T helper (Th)2 chemokines and improves the efficiency of Treg lymphocytes, while inhibiting the secretion of Th1 and Th17 chemokines. VitD plays a crucial role in maintaining normal immune function and facilitating communication between the innate and adaptive immune systems [27]. In the context of inflammatory bowel disease, VitD can also promotes macrophage M2 polarization [28], downregulate the activity of dendritic cells, induce tolerogenic T cell differentiation and function instead of pro-inflammatory responses, and increase the levels of anti-inflammatory chemokines through the activation of VDR, underscoring its significance [29]. In our study, we observed that PAAD cells overexpressing VDR promote the polarization of macrophages into the M2 phenotype, thereby contributing to the migration and recruitment of M2 macrophages. Macrophages are pivotal immune cells in PAAD, with the M2 phenotype playing a crucial role in immunosuppression, tumor progression, metastasis, and drug resistance (Fig. 8).

The interaction between immune cells and tumor cells relies on numerous chemokines. Our study revealed elevated expression of supernatant CCL20 in tumor cells, and the effect of overexpression of VDR tumors on macrophages disappeared after we blocked CCL20. This suggests that CCL20 may be a key molecule in regulating altered macrophages function. CCL20 also known as macrophage inflammatory protein 3α, is a small molecular weight cytokine of the CC chemokine family with strong chemotactic effects on lymphocytes. Esophageal cancer cells promote macrophage M2 polarization and recruitment through up-regulation of CSF1 and CCL20, respectively. While M2 macrophages promote tumor progression through FAK/PI3K/AKT after co-culture with tumor cells [30]. In a prostate cancer study, tumor cells can induce TAM infiltration and M2 polarization via the CCL20/CCR6 axis, leading to tumor progression [31]. It has been reported that VDR can act as a transcription factor to regulate the expression of several chemokines [32]. To test our hypothesis, we first applied the JASPAR database to predict that VDR has a binding site within the CCL20 promoter, which we subsequently confirmed using Chip and Luciferase reporter assays experiments, we also conducted mutation experiments to reconfirm that VDR binds to the promoter and regulates CCL20 transcription. Our study demonstrates for the first time that high VDR expression in PAAD cells promotes CCL20 transcription and release, inducing functional changes in tumor-associated macrophages.

We conducted a bioinformatics analysis and found that the high expression of VDR in PAAD is associated with a poor prognosis and the presence of immune cells, particularly an increased infiltration of M2 macrophages in the tumor tissue. This discovery provides a new avenue for investigating the limited therapeutic effectiveness of VDR agonists. Therefore, targeting macrophage function could be a potential approach to improve the treatment of PAAD using VDR agonists. However, there are certain limitations in this study. Firstly, our research solely focuses on the interaction between tumor cells and macrophages, disregarding the presence of other essential cells such as fibroblasts and pancreatic stellate cells in PAAD. These cells are also crucial to the development of PAAD [33, 34], and should be explored in future studies. Secondly, Numerous studies have demonstrated that the CCL20/CCR6 axis promotes macrophage M2 polarization and recruitment [30, 35], but further validation and exploration are lacking in this study. Tumor prevention can be achieved through VitD supplementation, but it is a double-edged sword for patients with advanced disease. Anti-macrophage therapy have achieved good therapeutic results clinically, and given the role of VDR on macrophage polarization and recruitment, administering VitD supplementation with anti-macrophage drugs to PAAD patients is expected to provide a new strategy for the clinical management.

### Electronic supplementary material

Below is the link to the electronic supplementary material.

Supplementary Material 1

Supplementary Material 2

## Data Availability

Data are available on reasonable request. All data relevant to the study are included in the article or uploaded as supplementary information.

## Abbreviations

PAAD: Pancreatic adenocarcinoma
VDR: Vitamin D receptor
VitD: Vitamin D
TAMs: Tumor-associated macrophages
MDSCs: Myeloid-derived suppressor cells
CAFs: Cancer associated fibroblasts
Tregs: Regulatory T cells
CRC: Colorectal cancer
OS: Overall survival
DFS: Disease-free survival
SsGSEA: Gene set enrichment analysis
SD: Standard deviation
DFI: Disease Free Interval
ChIP: Chromatin immunoprecipitation
