# Supplementary material for: VDR promotes pancreatic cancer progression in vivo by activating CCL20-mediated M2 polarization of tumor associated macrophage
Source: Cell Commun Signal. 2024 Apr 10;22:224. doi: 10.1186/s12964-024-01578-x (PMC11005177; doi:10.1186/s12964-024-01578-x)
Supplement: Supplementary file 1 — Supplementary Material 1 [file 12964_2024_1578_MOESM1_ESM.docx]

**Supplementary information**

**
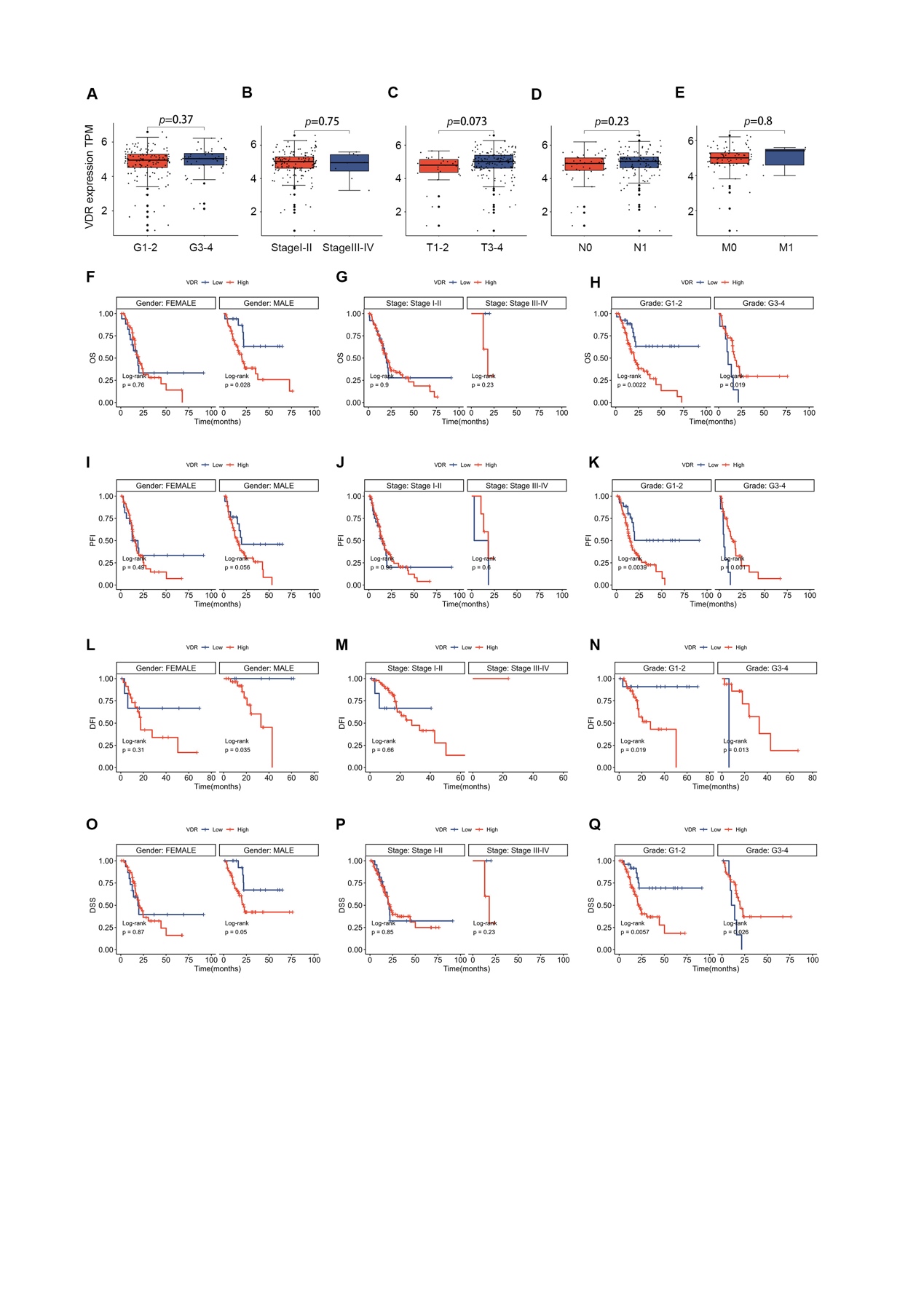
**

**Supplementary Figure.S1 Expression of VDR in different subgroups and prognostic curves**. (A-E) A correlation was observed between VDR expression with grade, stage, and TNM stage. (F-G) OS, PFI, DSS and DFI curves of the two groups with high and low VDR expression in different age, grade, and stage subgroups.


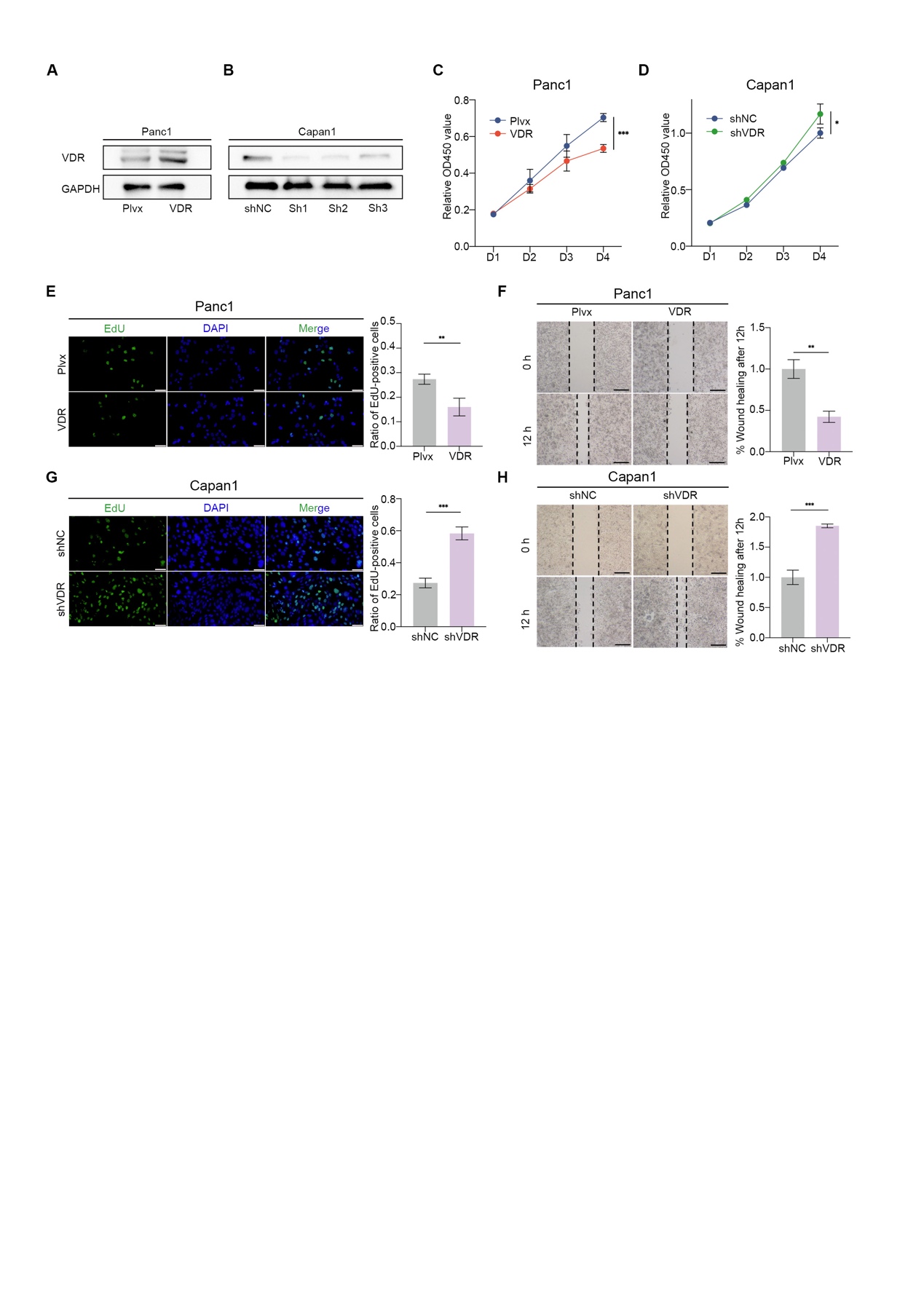


**Supplementary Figure.S2 Effect of VDR on PAAD proliferation and migration.** (A.B) Overexpression of VDR in Panc1 cells and knockdown of VDR in Capan1 cells. (C.D) Tumor cell viability after intervention of VDR expression. (E) EdU assay demonstrated that overexpression of VDR inhibited the proliferation of Panc1 cells. (F) while knocking down of VDR enhanced the proliferation of Capan1 cells. Scale bars=50μm. (G.H) Wound healing assay was performed on Panc1 cells and Capan1 cells, respectively. Results were measured after 24 hours. Scale bars=200μm.


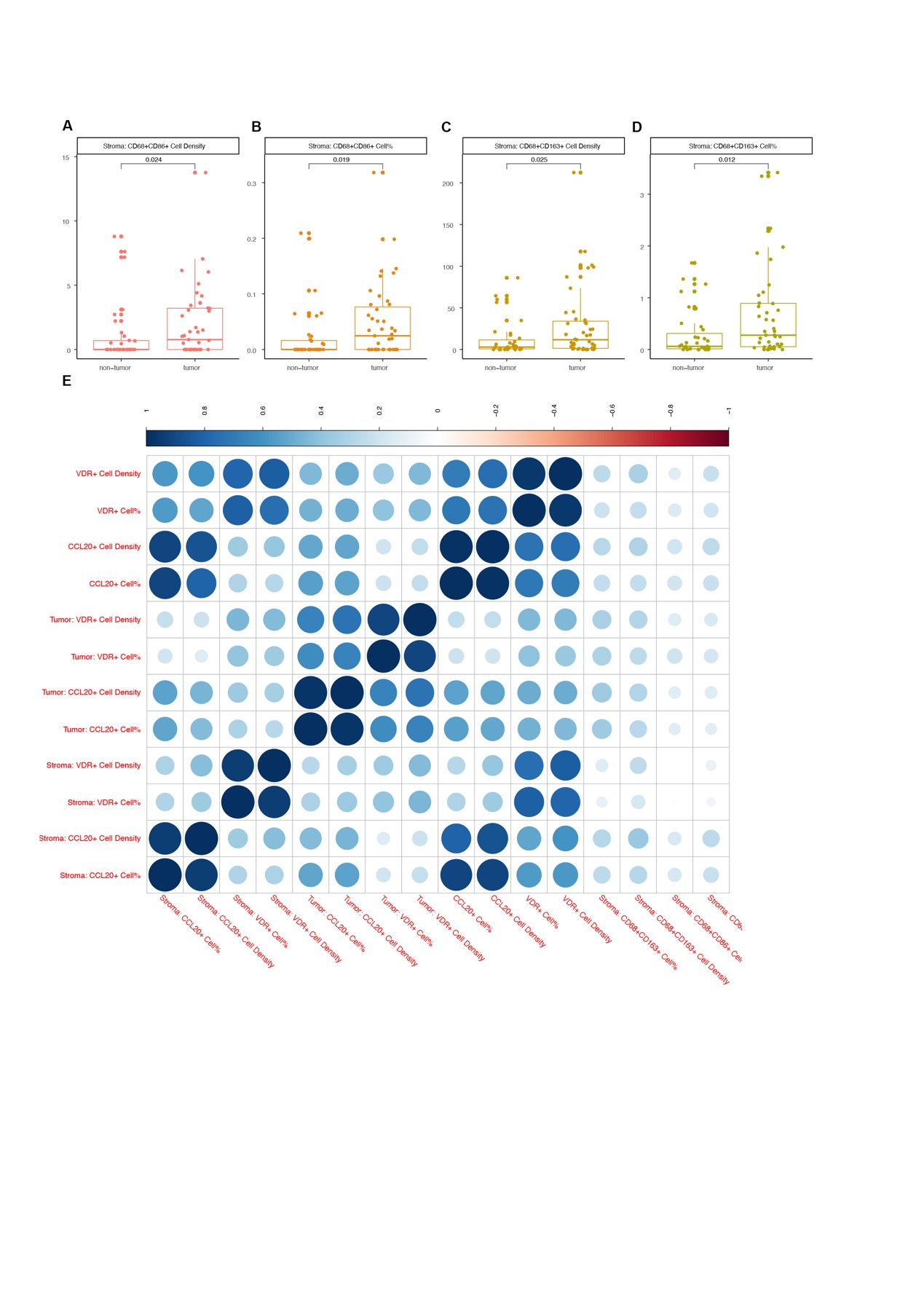


**Supplementary Figure.S3 Assessment of macrophage infiltration in tumor and paracancerous tissues.** (A-D) Initially, we observed that tumor tissues exhibited higher densities and proportions of both M1 and M2 macrophages compared to paraneoplastic tissues. (E) Correlation analysis of indicators.


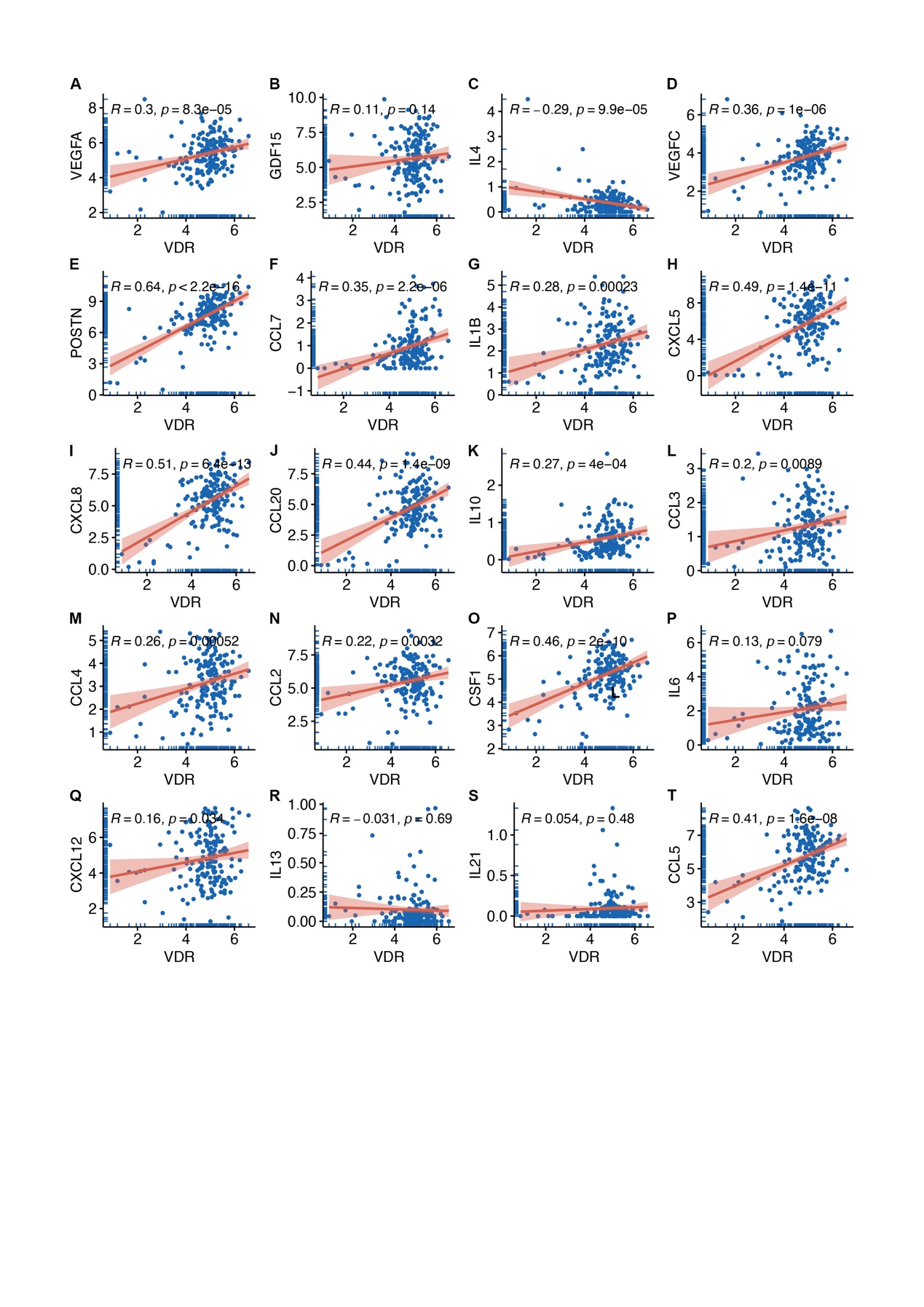


**Supplementary Figure.S4 Correlation of VDR expression with different cytokines in TCGA PAAD data.** (A-T) Correlation analysis of VDR expression with cytokines that promote macrophage polarization and recruitment.


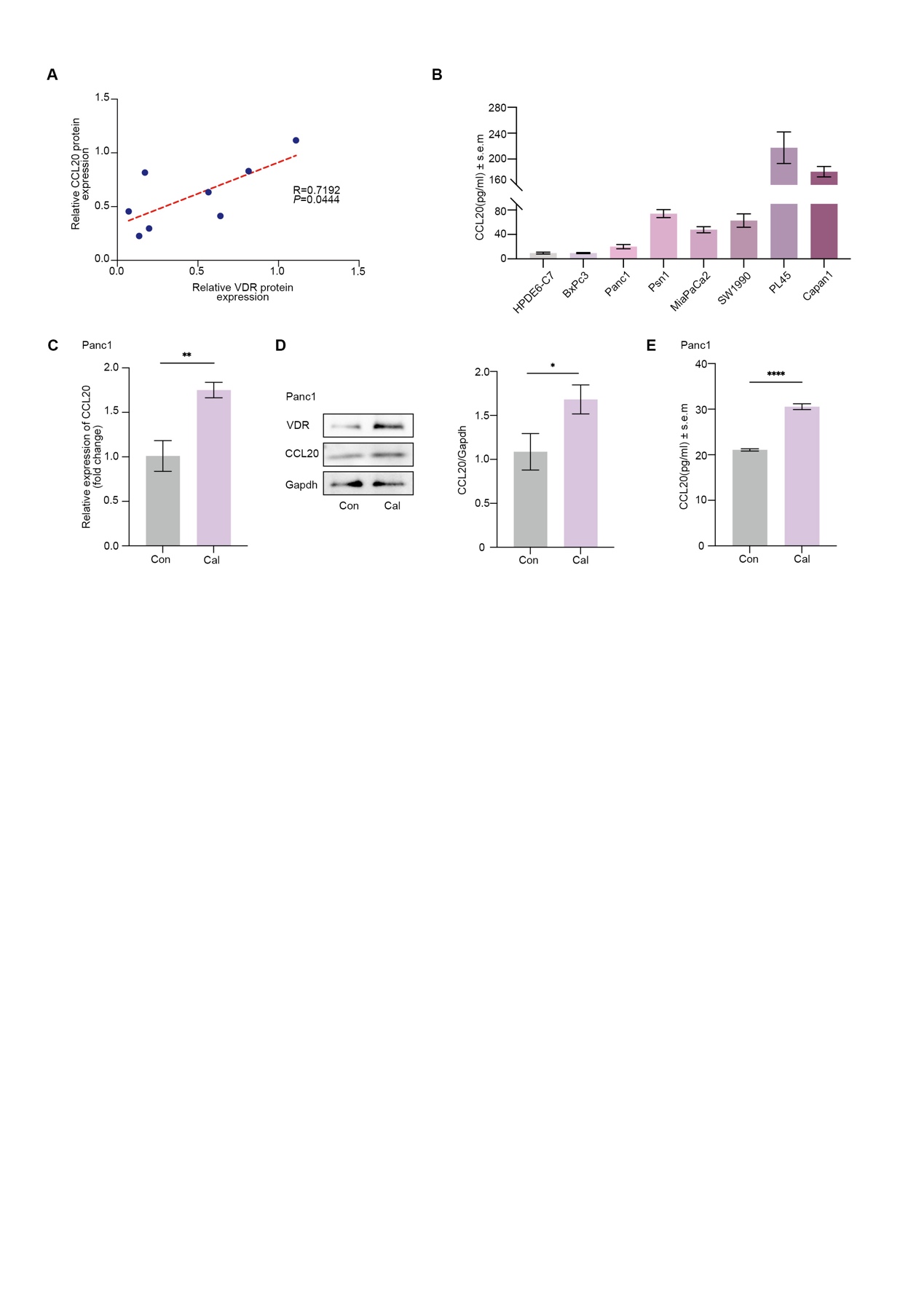


**Supplementary Figure.S5 Activation of VDR promotes CCL20 expression and release.** (A) Correlation of protein expression levels of VDR and CCL20 in PAAD cell lines. (B) Levels of CCL20 release in different PAAD cell lines. (C) Changes in CCL20 transcription and expression levels after activation of VDR using the VDR agonist calcitriol (Cal).
